# Comparative Epidemiology of Human Fatal Infections with Novel, High (H5N6 and H5N1) and Low (H7N9 and H9N2) Pathogenicity Avian Influenza A Viruses

**DOI:** 10.3390/ijerph14030263

**Published:** 2017-03-04

**Authors:** Zu-Qun Wu, Yi Zhang, Na Zhao, Zhao Yu, Hao Pan, Ta-Chien Chan, Zhi-Ruo Zhang, She-Lan Liu

**Affiliations:** 1 Department of Respiratory Medicine, the Second Affiliated Hospital, Zhejiang University School of Medicine, Hangzhou 310009, China; wuzuqun522@sohu.com; 2 Department of Medicine, Jinxi Petrochemical Hospital, Huludao 125001, China; zgdzy_zgdzy@sina.com; 3 National Research Center for Wildlife Borne Diseases, Key Laboratory of Animal Ecology and Conservation Biology, Institute of Zoology, Chinese Academy of Sciences, Beijing 100101, China; nazhao2007@163.com; 4 Department of Infectious Diseases and Key Lab of Vaccine against Hemorrhagic Fever with Renal Syndrome, Zhejiang Provincial Center for Disease Control and Prevention, Hangzhou 310051, China; zhy@cdc.zj.cn; 5 Department of Infectious Diseases, Shanghai Municipal Centre for Disease Control and Prevention, Shanghai 200336, China; scdcph@163.com; 6 Center for Geographic Information Science, Research Centre for Humanities and Social Science, Academia Sinica, Taipei 115, Taiwan; dachianpig@gmail.com; 7 School of Public Health, Shanghai Jiaotong University School of Medicine, Shanghai 200025, China

**Keywords:** influenza virus, endemic infection, horizontal transmission, seasonal incidence

## Abstract

This study aimed to assess the mortality risks for human infection with high (HPAI) and low (LPAI) pathogenicity avian influenza viruses. The HPAI case fatality rate (CFR) was far higher than the LPAI CFR [66.0% (293/444) vs. 68.75% (11/16) vs. 40.4% (265/656) vs. 0.0% (0/18) in the cases with H5N1, H5N6, H7N9, and H9N2 viruses, respectively; *p* < 0.001]. Similarly, the CFR of the index cases was greater than the secondary cases with H5N1 [100% (43/43) vs. 43.3% (42/97), *p* < 0.001]. Old age [22.5 vs. 17 years for H5N1, *p* = 0.018; 61 vs. 49 years for H7H9, *p* < 0.001], concurrent diseases [18.8% (15/80) vs. 8.33% (9/108) for H5N1, *p* = 0.046; 58.6% (156/266) vs. 34.8% (135/388) for H7H9, *p* < 0.001], delayed confirmation [13 vs. 6 days for H5N1, *p* < 0.001; 10 vs. 8 days for H7N9, *p* = 0.011] in the fatalities and survivors, were risk factors for deaths. With regard to the H5N1 clusters, exposure to poultry [67.4% (29/43) vs. 45.2% (19/42), *p* = 0.039] was the higher risk for the primary than the secondary deaths. In conclusion, old age, comorbidities, delayed confirmation, along with poultry exposure are the major risks contributing to fatal outcomes in human HPAI and LPAI infections.

## 1. Introduction

Avian influenza refers to the infection of birds with avian influenza type A viruses [1]. These viruses occur naturally among wild aquatic birds worldwide and can infect over 100 domestic sources of poultry as well as other birds and animal species [2,3,4,5,6,7]. Avian influenza viruses do not normally infect humans, but human infections may occur after contact with infected birds or their secretions or excretions, or through limited human-to-human transmission [8,9,10,11,12]. Given the significant global improvements in laboratory characterization and surveillance, additional novel avian viruses are likely to be identified. Following the appearance of the H5N1virus in 1997, ongoing surveillance efforts have already improved not only the detection of the H7N9 (in 2013), H10N8 (in 2013) and H5N6 subtypes (in 2014), which have all caused severe infections, but also the detection of other subtypes such as H6N1, H7N2, H7N3, H7N7, H9N2 and H10N7, which have resulted in mild infections in a limited number of humans [1,13,14].

Each new virus may have a distinct potential for animal-to-human transmission or to cause mild, severe or even fatal human illness. On the basis of the molecular characteristics of the viruses and their ability to result in disease and mortality in chickens in a laboratory setting, avian influenza A viruses have been classified into the following two categories: low pathogenic avian influenza (LPAI) A viruses and highly pathogenic avian influenza (HPAI A viruses [15]. The majority of those isolated have been LPAI A viruses, although HPAI A viruses have occasionally been detected. Notably, the case fatality rate (CFR) among human cases of avian influenza has ranged from 36%–60% overall, which is alarmingly high compared with all previous outbreaks of human cases of seasonal influenza in the United States, for which the CFR has ranged from 0.04%–1.0% [1,16,17]. This high level of illness severity and high mortality rate was unexpected and increased disease burden, resulting in concern among clinicians and public health officials; however, the risk factors that are most highly associated with the deaths from avian influenza were not clear.

On the basis of laboratory-confirmed deaths and the number of survivors, we examined human HPAI and LPAI infections in terms of the overall population, pediatric and clustered cases, with the aim of identifying the high-risk factors that are associated with fatal outcomes. This research will improve the clinical outcome and will also be helpful in decreasing the disease burden for these novel avian influenza viruses.

## 2. Materials and Methods

### 2.1. Ethical Statement 

The National Health and Family Planning Commission of China determined that the collection of data from human cases of avian influenza infection was part of the public health investigation of an outbreak and was exempt from institutional review board assessment. All other data were obtained from publicly available data sources. All data were supplied and analyzed in an anonymous format without access to personal identification information.

### 2.2. Study Populations, Case Definitions and Categorization

All laboratory-confirmed cases of infection with HPAI (H5N1 and H5N6) and LPAI (H7N9 and H9N2) in China were reported to the national system for reporting notifiable infectious diseases between 1 January 1997 and 30 November 2016. Other cases that occurred outside China were obtained from various publicly available sources, including local health authority news releases, ProMed posts, published literature and data reported to the World Health Organization (http://www.who.int/influenza/human_animal_interface/HAI_Risk_ Assessment/en/). A detailed distribution of these cases is shown in Table S1.

The HPAI and LPAI case definitions were determined on the basis of “the diagnosis and treatment programs of human infections with H7N9, H5N6, H9N2 and H5N1 viruses” issued by the National Health and Family Planning Commission of the People’s Republic of China [5,6,8,10,18].

A cluster was defined as two or more persons with an onset of symptoms within the same 14-day period, who were associated with a specific setting such as a classroom, workplace, household, extended family, hospital, other residential institution, military barracks, recreational camp or live bird market [19].

An index case is defined as the earliest identified occurrence of a disease or disorder, which usually emerges as part of an epidemiological investigation of a patient population or a genetic study of a family. The index case may indicate the source of the disease, the possible spread and the reservoir that holds the disease in between outbreaks. A secondary case is defined as one that occurs among the close contacts of a primary case within 14 days of the onset of illness in the primary patient [20].

### 2.3. Definitions of Exposure 

(1) Any exposure to poultry including: direct contact, indirect contact, proximity to healthy, sick or dead poultry (including all types of poultry or birds, e.g., chickens, ducks, geese, pet birds, pigeons, etc.), having poultry in the neighborhood and eating poultry products that have not been properly processed. (2) Visited live bird markets (LBMs): visiting an LBM in the two weeks prior to the onset of symptoms. LBM refers to any wholesale or retail market that sells live poultry or birds. (3) Exposure to sick or dead poultry: direct contact, indirect contact, and proximity to sick or dead poultry in the two weeks before the onset of symptoms. (4) Exposure to backyard poultry: poultry raised in an affected individual’s own backyard or neighborhood in the two weeks before the onset of symptoms. (5) Human case contact: close contact with a confirmed or probable case of human H5N1 (any time from the day before the onset of illness to the death of the affected individual, or during the period that this individual was hospitalized) in the two weeks before the onset of symptoms [10].

### 2.4. Epidemiologic Investigations

Provincial epidemiologists and local public health doctors conducted face-to-face interviews with all affected individuals, their family members and medical staff using a standard questionnaire designed by the Chinese Center for Disease Control and Prevention (China CDC). A variety of epidemiologic information was collected, including that which is related to personal information, comorbidity, and exposure condition and infection areas. Investigations generally began within 24 h of a diagnosis of suspected infection, clinical circumstances permitting.

A standardized case history form and an additional medical chart, including information regarding dates of illness onset, hospital admission, death or discharge and antiviral treatment, was prepared and completed by frontline physicians in the local hospitals responsible for the diagnosis and outcome of avian influenza cases. All of the surveyors were thoroughly trained in the survey procedure and instrument to ensure that they conducted the interviews according to uniform standards and methods. 

### 2.5. Laboratory Tests

All of the Chinese cases of avian influenza were obtained as patient respiratory specimens, which were shipped to the local hospitals and local CDC at 4 °C for laboratory testing for H5N1 [21], H5N6, H7N9 and H9N2 using reverse transcription polymerase chain reaction (rRT-PCR) [22,23]. All of the surveyors and laboratory technicians were thoroughly trained to ensure that the interviews and laboratory investigations were conducted according to uniform standards. 

Total viral RNA was extracted from the respiratory specimens using a Qiagen RNeasy Mini Kit, according to the manufacturer’s instructions. The specific primer and probe sets were provided by the China CDC. All cases were confirmed by rRT-PCR methods.

### 2.6. Data Statistical Analysis

We plotted the geographical locations of people infected with the four viruses, and the current locations of all confirmed cases were geocoded by the Google Map geocoding service (https://google developers.appspot.com/maps/documentation/javascript/examples/geocoding-simple). After obtaining the X (longitude) and Y (latitude) coordinates, we used ArcGIS version 10.2 (ArcGIS, Redlands, CA, USA). The world basemap, which is publicly available and maintained by Environmental Systems Research Institute, Inc. (ESRI, ArcGIS, Redlands, CA, USA) (http://www.arcgis.com/home/item.html?id=3864c63872d84aec91933618e3815dd2), was used for the spatial analysis and for constructing a spatial distribution map of the cases. Second, comparative epidemical analyses of the dates of the onset of illness and the characteristics of the HPAI and LPAI fatalities and survivors were conducted. 

All statistical analyses were conducted using the Statistical Analysis System, version 9.2 (SAS Institute, Cary, NC, USA). Quantitative measurements are presented as the median and range of the observed values, and qualitative measurements are presented as relative and absolute frequencies. ANOVA analysis was used to measure the data over the H5N1, H5N6, H7N9 and H9N2 groups, and a T-test was used to analyze the differences between the fatality and survivor groups. Chi-square tests (*x*^2^) were used to compare the distribution of the different variables of qualitative measurements between the fatality and survivor groups. Fisher’s exact test was used in the analysis of contingency tables when the sample sizes were small [the expected values in any of the cells of a contingency table were below 5; the number of total samples was no more than 40; the data were very unequally distributed among the cells of the table]. Any *p* values given are two-sided, and were considered statistically significant at 0.05.

## 3. Results

### 3.1. Epidemiological Findings in the HPAI (H5N1 and H5N6) and LPAI (H7N9 and H9N2) Fatalities and Survivors in the Overall Population

#### 3.1.1. Overall Case Fatality Rate (CFR)

**Figure 1:**
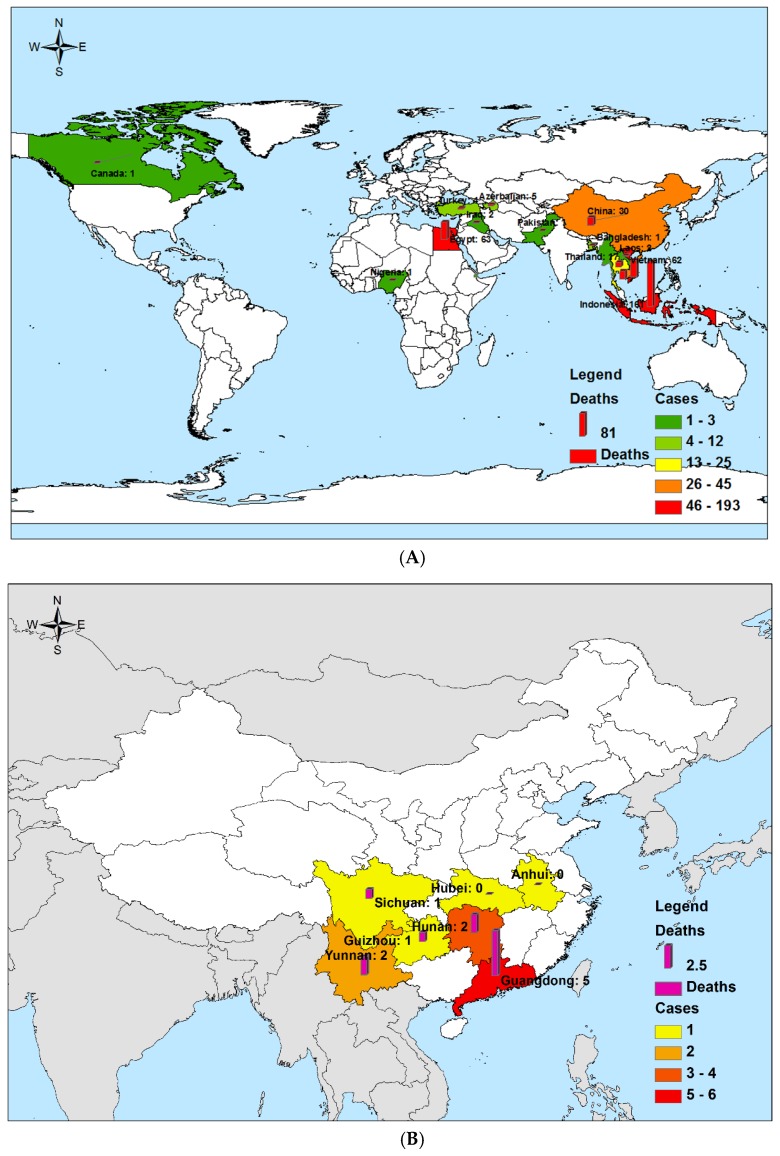

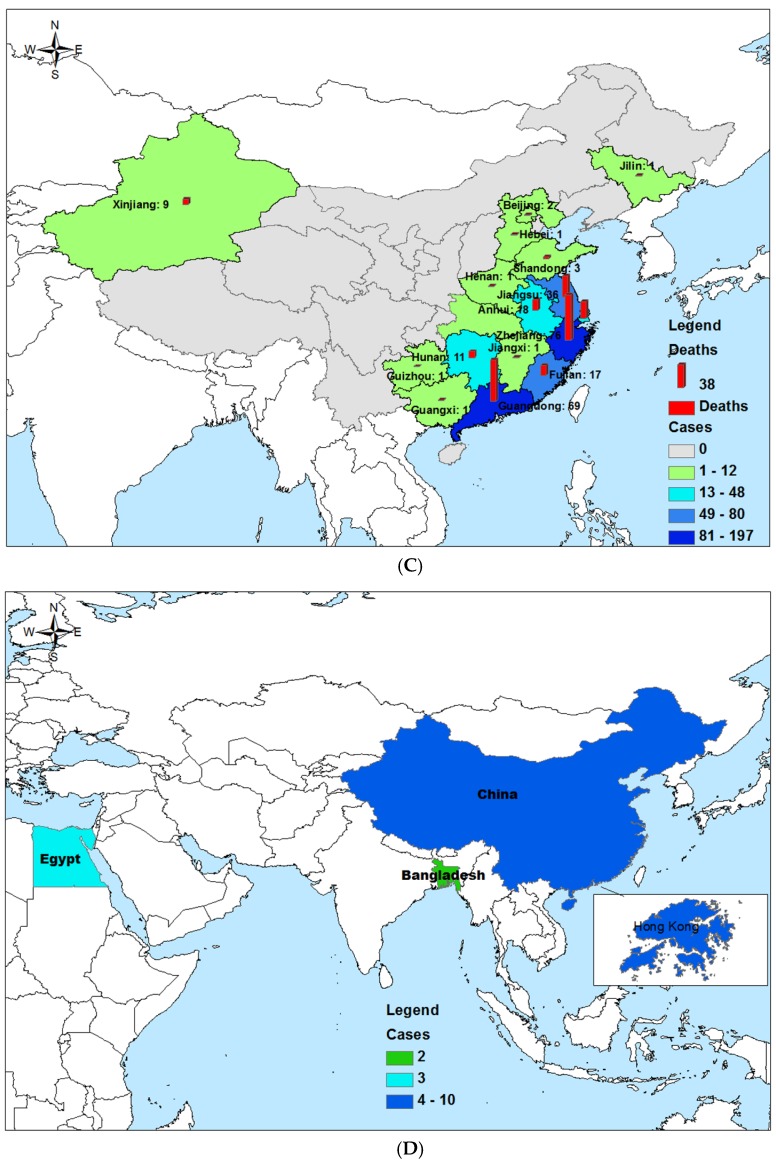
The geographic distribution of the total number of highly pathogenic avian influenza A (H5N1 and H5N6) and low pathogenic A (H7N9 and H9N2) virus cases and deaths until November of 2016. The shadow and the bar in the map were generated by the number of total cases and the deaths, respectively. (**A**) Highly pathogenic avian influenza A (H5N1): total human cases (N = 638) and deaths (N = 379). (**B**) Highly pathogenic avian influenza A (H5N6): total human cases (N = 16) and deaths (N = 11). (**C**) Low pathogenic avian influenza A (H7N9): total human cases (N = 676) and deaths (N = 275). (**D**) Low pathogenic avian influenza A (H9N2): total human cases (N = 21) and deaths (N = 0).

A series of laboratory-confirmed HPAI (H5N1 and H5N6) and LPAI (H7N9 and H9N2) fatalities and survivors were analyzed (Table 1). We obtained data relating to a total of 444 HPAI H5N1 cases (293 fatalities and 151 survivors) that were globally reported between 1 January 1997 and 30 November 2016 (Figure 1a, Table 1). We also obtained data regarding 16 laboratory-confirmed human cases of HPAI H5N6 (5 survivors and 11 fatalities) reported in China since the first case was confirmed on 3 May 2014, up until 30 November 2016 (Figure 1b, Table 1). We selected data relating to 656 LPAI laboratory-confirmed H7N9 cases reported (265 fatalities and 391 survivors), since the first case was confirmed in China on 30 March 2013, up until 30 November 2016 (Figure 1c, Table 1), and then chose 18 LPAI laboratory-confirmed H9N2 cases (all survivors) that had been reported since the first case was confirmed in China in 1998, up until 30 November 2016 (Figure 1d, Table 1). The CFR for HPAI H5N1 [66.0% (293/444)] and H5N6 cases [68.75% (11/16)] was statistically significantly higher than those for LPAI H7N9 [40.4% (265/656)] and H9N2 cases [0.0% (0/18)] (*p* < 0.001) (Table 1).

#### 3.1.2. Diseases Distribution

Fatal H5N1 cases were reported in 87.5% (14/16) of the countries in which it had been found (Figure 1a, Table 1), fatal H5N6 cases were reported in 71.43% (5/7) of the identified Chinese provinces (Figure 1b, Table 1), and H7N9 cases were reported from 95.0% (19/21) of the reporting Chinese provinces (Figure 1c, Table 1). No fatal H9N2 cases were reported in Bangladesh, China, Egypt or Hong Kong Special Administrative Region (Figure 1d, Table 1).

**Figure 2:**
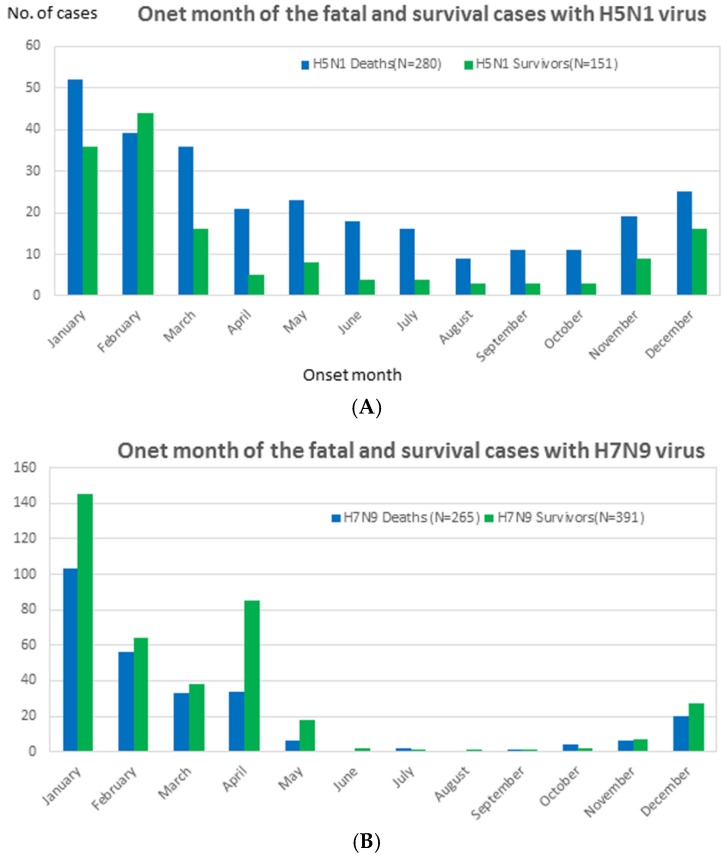
The onset month distribution of highly pathogenic avian influenza A (H5N1) and low pathogenic A (H7N9) virus fatalities and survivors until November 2016. (**A**) The onset month distribution of highly pathogenic avian influenza A (H5N1): survivors (N = 280) and deaths (N = 151). (**B**) The onset month distribution of low pathogenic A (H7N9) viruses: survivors (N = 265) and deaths (N = 391).

Four HPAI (H5N1 and H5N6) and LPAI (H7N9 and H9N2) viruses circulate in a full year, peaking during the winter and spring and occurring annually from November through to April, in particular. We observed no differences between the number of fatalities and survivors according to the seasonal distributions of H5N1 or H7N9 infections (Figure 2a,b).

**Figure 3:**
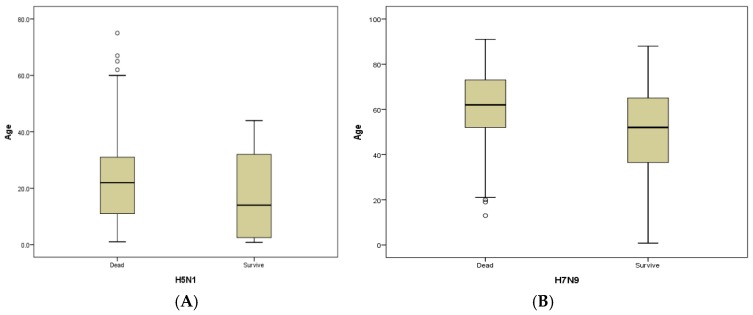
A detailed boxplot figure for the age of the fatal and survival cases with highly pathogenic avian influenza viruses H5N1 and low pathogenicity avian influenza viruses H7N9 virus dates of onset from 1 January 1997 to 30 November, 2016. (**A**) The age distribution of highly pathogenic avian influenza A (H5N1): survivors (N = 151) and deaths (N = 293). (**B**) The age distribution of low pathogenic avian influenza A (H7N9): survivors (N = 391) and deaths (N = 265).

The median age of people who died of the H7N9 and H5N1viruses was much higher than that of those who survived. In the H5N1 groups, the median age of those who died was 22.5 (1–75) vs. 17 (8 months–75 years) years for those who survived (*p* = 0.018). In the H7N9 groups, the median age of those who died was 61 (13–91) vs. 49 (8 months–88 years) years for those who survived (*p* < 0.001); the median age of those who died of the H5N6 virus was slightly older than that of those who survived [39 (25–50) vs. 35 (5.5–65) years, respectively (No *p* value is available for such small groups)]. The median age of the H9N2 survivors was 13 years (9 months–86 years), which was the youngest of those who survived these four viruses. In general, the median age of the H7N9 fatalities [61 years (13–91)] and survivors [49 (8 months–88 years)] was much higher than that of the H5N1 fatalities [22.5 years (1–75)] and survivors [17 years (8 months–75 years] [*p* < 0.001 for all] (Table 1). The predominant age for fatalities was identified as being 20–29, 40–49 and over 60 years in the H5N1, H5N6 and H7N9 groups, respectively [*p* < 0.001] (Table 1, Figure 3).

The gender characteristics of the HPAI cases are unusual, compared with those of the LPAI cases. There was a similar gender distribution of H5N1 fatalities and survivors [43% (126/293) vs. 42.3% (41/97) male cases, *p* = 1.000], whereas a higher male distribution of H5N6 fatalities and survivors was observed [45.45% (5/11) vs. 40.00% (2/5)]; no difference in *p* value due to a small sample size] across the HPAI cases; however, in the LPAI cases, the male population was primarily affected with the H7N9 virus: 70.1% (183/261) of fatalities vs. 68.8% (267/388) of survivors, respectively, *p* = 0.795]. In contrast, males accounted for only 33.3% (5/15) of survivors of the H9N2 virus. In total, there was no difference in the gender distribution between the H5N1 and H7N9 fatalities and those who survived; however, males were the predominant population with regard to H7N9 fatalities [70.1% (183/261)] and survivors [68.8% (267/388)], compared with H5N1 fatalities [42.7% (126/295)] and survivors [42.3% (41/97)] (*p* < 0.001 for all) (Table 1).

#### 3.1.3. Exposure History

A history of exposure to poultry prior to onset was common for both the H5N1 fatality and survival groups (Table 1); however, poultry exposure history was not statistically significantly different between the two groups, with the exception of visiting LBMs for the H7N9 group (*p* = 0.011). Exposure to sick or dead poultry [37.5% (30/80) in the H5N1 group vs. 5.9% (12/205) in the H7N9 group, *p* < 0.001] and to backyard poultry [25.0% (20/80) in the H5N1 group vs. 6.8% (14/205) in the H7N9 group, *p* < 0.001] was more often observed in the H5N1 fatality groups than in the H7N9 fatality groups; however, visiting LBMs was less commonly reported in the H5N1 groups than in the H7N9 groups [7.5% (6/80) vs. 62.9% (129/205), respectively, *p* < 0.001]. All of the H5N6 cases and 77.8% (7/9) of the H9N2 cases had a history of poultry exposure (Table 1). We stratified exposure to poultry by gender in the H5N1 and H7N9 groups, and found that there were no gender biases.

### 3.2. Clinical Findings in the HPAI (H5N1 and H5N6) and LPAI (H7N9 and H9N2) Fatalities and Survivors in the Overall Population

#### 3.2.1. Comorbidity 

The ratio of comorbidity was much higher in the H5N1 and H7N9 virus fatalities than in the survivors [18.8% (15/80) vs. 8.33% (9/108), *p* = 0.046 for H5N1; 58.6% (156/266) vs. 34.8% (135/388), *p* < 0.001 for H7N9]. Only two H5N6 survivors were found to have underlying conditions, one of which was pregnancy, while only 22.2% (2/9) of the H9N2 survivors had comorbidities. In total, the rate of comorbidities in the H7N9 fatality and survivor groups was slightly higher than that of the H5N1 groups [*p* < 0.001] (Table 1).

#### 3.2.2. The Clinical Period

**Figure 4:**
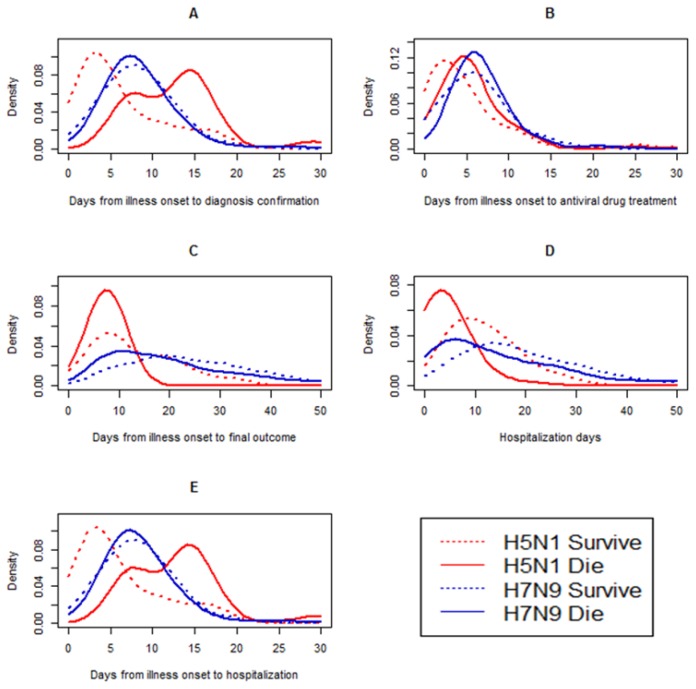
Comparisons of time-delay distributions for laboratory-confirmed human avian influenza A H7N9 and H5N1 virus fatalities and survivors. The number of H5N1 fatalities = 280; the number of H5N1 survivors = 151; the number of H7N9 fatalities = 265; and the of H7N9 survivors = 391. (**A**) Days from illness onset to laboratory confirmation of influenza A H7N9 or H5N1 virus fatalities and survivors. (**B**) Days from illness onset to antiviral treatment of influenza A H7N9 or H5N1 virus fatalities and survivors. (**C**) Days from illness onset to final outcome of infection (fatality or survival) with influenza A H7N9 or H5N1 viruses. (**D**) Days of hospitalization for A H7N9 or H5N1 fatalities and survivors. (**E**) Days from illness onset to hospitalization for A H7N9 or H5N1 fatalities and survivors.

Five time periods that are useful for public health surveillance were evaluated. For the H5N1 group, with the exception of the median days from onset to antiviral treatment, there were differences between the fatalities and survivors in other median days, including days from onset to hospitalization [5.5 (0–20) vs. 5 (0–31) days, *p* = 0.023]; days from onset to confirmation of infection [13 (6–29) vs. 6 (2–17) days, *p* < 0.001]; days from onset to outcome [10 (2–27) vs. 13 (3–33) days, *p* = 0.019]; and days of hospitalization after onset [4 (0–26) vs. 11 (6–27) days, *p* = 0.001] (Table 1, Figure 4).

For the H5N6 group, the number of median days was similar to those of the H5N1 fatalities and survivors (we could not perform statistical analyses on all seven cases) (Table 1, Figure 4). 

For the H7N9 group, the median number of days from onset to confirmation of infection in the fatality groups was slightly longer than that of survivors [10 (1–51) vs. 8 (1–28) days, *p* = 0.011]; however, the median number of days from onset to outcome [23 (3–111) vs. 31 (4–187) days, *p* < 0.001] and number of hospitalization days [18 (0–103) vs. 25 (1–179) days, *p* < 0.001] in the fatality groups was slightly less than those relating to survivors, respectively (Table 1, Figure 4).

The number of days from onset to confirmation of H9N2 infection in survivors was 17 (2–43) days, and this number was close to those in H9N2 infection cases (17 days) (Table 1, Figure 4). There were statistical differences in the numbers of the other four median day variables, with the exception of the number of days from onset to hospitalization identified in the H5N1 and H7N9 fatalities and survivors (*p* < 0.05 for all) (Table 1, Figure 4).

### 3.3. Comparative Epidemiology of the Fatalities and Survivors of HPAI (H5N1) and LPAI (H7N9) in Children (<15 Years Old)

#### 3.3.1. CFR in Children

Far higher numbers of children died in the H5N1 group than in the H7N9 group [33.1% (97/293) vs. 0.4% (1/265), *p* = 0.030], and the CFR was much higher in the H5N1 group than in the H7N9 group [42.5% (97/228) vs. 2.4% (1/41), respectively, *p* < 0.001].

#### 3.3.2. Age and Gender Distribution

The mean age of pediatric death was 6 (0.9–15) years in the H5N1 group, which is significantly higher than that of those who survived [4 (0.7–15) years, *p* < 0.001]. In contrast, no difference in the median age was found between the H5N1 and H7N9 virus survivors [5.0 (0.75–15) years, *p* = 0.153] (Table 2). There was no significant difference between the H5N1 fatality and survivor groups in the percentage of male children [46.4% (45/97) vs. 51.9% (68/131), *p* = 0.410]. The same was observed in the H5N1 and H7N9 survivor groups [51.9% (68/131) vs. 45.2% (19/42), *p* = 0.452] (Table 2). 

### 3.4. Clinical Findings of the Fatalities and Survivors of HPAI (H5N1) and LPAI (H7N9) in Children (<15 years old)

In the H5N1 groups, the median number of days from onset to confirmation of infection was much higher in the fatality group than in the survival group [10 (3–15) vs. 3 (3–14) days, *p* = 0.034], as was the median number of days to antiviral treatment [7 (0–14) vs. 4 (0–25) days, *p* = 0.044] (Table 2). In addition, the median number of days from onset to hospitalization was different in the H5N1 and H7N9 survivor groups [6 (0–25) days vs. 2.0 (0–8) days, *p* = 0.008], as was the median number of days to confirmation of infection [3 (3–14) vs. 6.5 (1–67) days, *p* = 0.025] and to antiviral treatment [4 (0–25) vs. 2.5 (0–13) days, *p* = 0.045] (Table 2).

### 3.5. Comparative Epidemiology of the Index and Secondary Deaths in the Clustered HPAI (H5N1) and LPAI (H7N9) Cases

#### 3.5.1. CFR in the Clustered Cases

In the H5N1 group, the CFR was statistically significantly higher in the index fatalities than in the secondary fatalities [100% (43/43) vs. 43.3% (42/97), respectively, *p* < 0.001], as was the number of people with comorbidities [9.3% (4/43) vs. 0.0% (0/42), respectively, *p* = 0.043]; however, there were no differences between H7N9 virus index and secondary fatalities in the CFR and underlying diseases (Table 3).

#### 3.5.2. Exposure History

The rate of poultry exposure was far higher for the index fatalities than for the secondary fatalities with regard to both the H591 virus [67.4% (29/43) vs. 45.2% (19/42), respectively, *p* = 0.039] and for the H7N9 virus [100% (9/9) vs. 50% (3/6), respectively, *p* = 0.018]; however, common exposure or human case contact was slightly lower for the index than for the secondary H5N1 fatalities [0.0% (0/43) vs. 28.6% (12/42), respectively, *p* < 0.001], with the same being observed with regard to H7N9 [11.1% (1/9) vs. 100% (6/6) in the index and secondary deaths for H7N9, *p* = 0.001] (Table 3). The results showed that there were no differences in the percentage of total deaths, the mean age, gender distribution or the median days between the index and secondary deaths with regard to the two viruses (Table 3).

### 3.6. Multivariate Logistic Regression Model Assessing Odds Ratios of Risk for Death for Case-Patients Infected with Highly Pathogenic Avian Influenza (H5N1) and Low Pathogenicity Avian Influenza (H7N9) Virus

Univariate logistic regression models for each risk factor showed that older age, having a concurrent health condition, exposure to poultry, delayed confirmation and antivirus treatment, were associated with death caused by H7N9 and H5N1 virus (all *p* < 0.05). Four variables remained significant after we adjusted for all 5 variables in a multivariate logistic regression model except the median days from onset to antivirus treatment in H5N1 group and only one variable related with the risk for the deaths in H7N9 group (Table 4). However, a male patient seems to increase odds of death in H7N9 groups, this relationship was not significant. This suggested the gender was not an indicator for death.

## 4. Discussion

Previous studies have shown that the severities of the illnesses caused by avian influenza are linked to host factors, including chronic diseases, immuno-suppressive disorders, delayed confirmation of infection and late antiviral treatment [5,24,25,26,27], as well as virus factors such as pathogenicity, replications and mutations [25,28]. In the present study, we aimed to identify the high risks associated with host factors with regard to fatal outcomes. 

In accordance with the results of a previous study, the CFR for overall HPAI cases was much higher than for LPAI [1]. The final H5N1 CFR may be reasonably estimated, because asymptomatic or mild human influenza A (H5N1) virus infection is rare [29]. In contrast, the H7N9 CFR might be overestimated, because a large number of mild cases in younger people are likely to go undetected [30,31]. Similarly, the final CFR for children in the H5N1 group was significantly greater than in the H7N9 group in the present study. Several studies have reported that 22 mild pediatric cases of infection with H7N9 viruses have been identified through the sentinel surveillance of influenza-like-illness and have had good outcomes [32,33]. Contributing to these findings was the fact that the majority of the children were secondary cases under medical investigation, which led to earlier confirmation and anti-viral drug treatment [34]. In general, CFR variation in different subtypes and different populations has been influenced by host features such as age, exposure history, medical-seeking behavior and underlying diseases [35,36].

Same to the previous reports on H1 and H3 seasonal influenza, older age and preexisting concurrent health conditions have been associated with an increase in the chance of death from high and low pathogenic avian influenza [37]. The median age and comorbidities of people who died from H5N1 and H759 infection were much greater compared with survivors [5]; however, there was no difference in gender distribution between the fatality and survivor groups. The predominance of an aged population has been attributed to higher incidences of underlying diseases and impaired immune functions, which may increase the susceptibility and progression of the infections and even increase the number of deaths [38].

A history of exposure to poultry prior to onset was not strongly related to fatal outcomes; however, there were statistical differences in exposure history in the HPAI and LPAI fatalities. The visited LBMs variable was much higher in the H7N9 fatality group than in the H5N1 fatality group. In contrast, the proportions of the exposure to sick and dead birds and backyard birds in H5N1 fatalities were much higher than the H7N9 fatality groups. These data suggest that contamination of LBMs and bird-to-bird transmission of H7N9 in these markets may be the primary initial mechanisms for increasing the transmission of the virus [39]. In contrast, H5N1 circulates in wild birds and infects poultry in backyards and small farms, as well as sick and dead birds [1,29]. These findings indicate that live and backyard birds are an ongoing source of exposure for birds and humans and represent a group in which HPAI and LPAI control could be implemented [1].

The clinical course of fatal and nonfatal infections of HPAI does not follow the typical pattern of LPAI infection. The median number of days from onset to confirmation of infection was clearly longer in the H5N1 and H7N9 virus fatality and survivor groups, indicating that delayed confirmation contributed to fatal outcomes. This is consistent with other retrospective studies of avian influenza A virus infections [40]. In particular, the period from onset to antiviral treatment in the children who died was also much longer than in those who survived, indicating that delayed oseltamivir treatment was a high-risk factor for H5N1, which is in accordance with previous reports on influenza viruses [41,42]. 

Among the well-described clusters of HPAI and LPAI avian influenza, it appears that the secondary cases were less severe than the index cases [9,11,12,43,44,45]. Similar to other research, the secondary deaths occurring in the H5N1 virus case clusters were markedly less severe than the index fatalities. There were several reasons for an important case ascertainment bias. On one hand, the secondary cases were detected, and the antiviral treatment began early through the healthcare surveillance system, and on the other, the index cases were biased toward older people with a higher number of underlying diseases [46]. In addition, the index cases generally had significantly higher levels of exposure to poultry, while the secondary cases subsequently became infected after providing care for the ill index patients or after spending prolonged periods of time with them. These findings show that poultry exposure and comorbidities are the major risks of death from the H5N1 virus.

## 5. Conclusions

In summary, the data from the present study suggest that the HPAI CRF is biased upward compared to all symptomatic LPAI cases in the overall and pediatric populations. This suggests that the severity and disease burden of HPAI is significantly higher than that of LPAI. Aged people, a greater number of concurrent health conditions, delayed confirmation of infection, and delayed antiviral drug treatment were the major factors contributing to a higher risk of deaths from HPAI and LPAI, whereas exposure to poultry and gender had no clear link with the fatalities. In contrast, the H5N1 CFR index cases were much higher than that of secondary cases. Although the reasons for this are not understood, they have been attributed to greater incidences of underlying diseases and poultry exposure history in the index cases. 

Therefore, to decrease future HPAI and LPAI mortality, it is necessary to develop early and rapid detection and to begin antiviral treatment as soon as possible. In addition, exposure to poultry must be decreased, especially for high-risk groups. These findings, providing all mortality-related risks for HPAI and LPAI, suggest that effective methods to reduce morbidity, mortality, and the corresponding disease burden, must be adopted.

## Figures and Tables

**Table 1:** Epidemical and clinical comparison of highly pathogenic avian influenza and low pathogenicity avian influenza fatalities and survivors.

Characteristics	HPAI					L PAI					*p3*	*p4*
	H5N1			H5N6		H7N9			H9N2			
	Fatalities (N = 293)	Survivors (N = 151)	*p1*	Fatalities (N = 11)	Survivors (N = 5)	Fatalities (N = 265)	Survivors (N = 391)	*p2*	Fatalities (N = 0)	Survivors (N = 18)		
**Epidemical characteristics**												
**CFR [% (No.)]**	66.0 (293/444)		-	68.75 (11/16)		40.4 (265/656)		-	0.0 (0/18)		<0.001	-
**Percentage of countries/provinces reporting fatalities [% (No.)]**	87.5 (14/16)		-	71.43 (5/7)		95.0 (19/21)		-	0.0 (0/4)		0.587	-
**Reported onset date of the first fatality**	2003/11/25		-	2014/4/23		2013/3/3		-	1998		-	-
**Reported date of last fatality**	2015/1/12		-	2016/11/20		2015/5/28		-	-		-	-
**Peak season**	January	February	-	January	December	January	January	-	-	December	-	-
**Exposure history [% (No.)]**												
**Any exposure to poultry (Total)**	91.25 (73/80)	95.4 (103/108)	0.366	100 (11/11)	100 (5/5)	49.3 (101/205)	55.1 (140/254)	0.223	-	77.8 (7/9)	<0.001	<0.001
**Males**	93.0 (40/43)	92.6 (38/41)	1.0000	-	-	37.2 (45/121)	47.3 (53/112)	0.1175		-	-	-
**Females**	89.2 (33/37)	97.0 (65/67)	0.1828	-	-	44.8 (22/49)	50.0 (26/52)	0.6078		-	-	-
**Exposure to sick or dead poultry**	37.5 (30/80)	25.0 (27/108)	0.196	27.3 (3/11)	0.0 (0/5)	5.9 (12/205)	3.9 (10/254)	0.379	-	11.1 (1/9)	<0.001	<0.001
**Backyard poultry**	25.0 (20/80)	17.6 (19/108)	0.275	0.0 (0/11)	0.0 (0/5)	6.8 (14/205)	9.1 (23/254)	0.491	-	0.0 (0/9)	<0.001	0.030
**Visited LBM**	7.5 (6/80)	8.3 (9/108)	1.000	63.64 (7/11)	60 (3/5)	62.9 (129/205)	50.8 (129/254)	0.011	-	44.4 (4/9)	<0.001	<0.001
**Human case contact**	1.25 (1/80)	0.9 (1/108)	0.486	0.0 (0/11)	0.0 (0/5)	3.9 (8/205)	7.1 (18/254)	0.160	-	0.0 (0/9)	1.000	0.074
**Unknown**	1.25 (1/80)	5.6 (6/108)	-	9.09 (1/11)	40 (2/5)	10.7 (22/205)	7.9 (20/254)	-	-	0.0 (0/9)	-	-
**Comorbidity [% (No.)]**	18.8 (15/80)	8.33 (9/108)	0.046	36.36 (4/11)	0.40 (2/5)	58.6 (156/266)	34.8 (135/388)	<0.001	-	22.2 (2/9)	<0.001	<0.001
**Gender [Male% (No.)]**	43 (126/293)	42.3 (41/97)	1.000	45.45 (5/11)	40.00(2/5)	70.1 (183/261)	68.8 (267/388)	0.795	-	33.3 (5/15)	<0.001	<0.001
**Median age (Range, Years)**	22.5 (1–75)	17 (8 months–75 years)	0.018	39 (25–50)	35 (5.5–65)	61 (13–91)	49 (8 months–88 years)	<0.001	-	13 (9 months–86 years)	<0.001	<0.001
**Age group [No. (%),(Years)]**												
0–9	63 (22)	42 (43)	<0.001	0 (0)	1 (20)	0 (0)	38 (10)	<0.001	-	11 (79)	<0.001	<0.001
10–19	65 (22)	14 (14)		0 (0)	1 (20)	2 (1)	7 (2)		-	1 (7)		
20–29	76 (26)	10 (10)		2 (18.18)	0 (0)	10 (4)	20 (5)		-	-		
30–39	62 (21)	15 (15)		2 (18.18)	1 (20)	21 (8)	54 (14)		-	-		
40–49	17 (6)	13 (13)		6 (54.55)	0 (0)	21 (8)	48 (12)		-	1 (7)		
50–59	5 (2)	1 (1)		1 (9.09)	1 (20)	60 (23)	92 (24)		-	-		
Over 60	5 (2)	2 (2)		0 (0)	1 (20)	151 (57)	128 (33)		-	1 (7)		
**Median number of days**												
**Days from onset to hospitalization**	5.5 (0–20)	5 (0–31)	0.023	4 (0–7)	4.5 (3–6)	5 (0–31)	5 (0–28)	0.761	-	2 (1–5)	0.954	0.071
**Days from onset to confirmation of infection**	13 (6–29)	6 (2–17)	<0.001	13 (5–20)	15 (10–20)	10 (1–51)	8 (1–28)	0.011	-	17 (2–43)	0.027	0.020
**Days from onset to antiviral treatment**	6 (0–14)	5 (0–31)	0.202	9 (1–14)	7 (0–12)	7 (0–23)	6 (0–19)	0.089	-	-	<0.001	0.020
**Days from onset to outcome**	10 (2–27)	13 (3–33)	0.019	8 (4–10)	58	23 (3–111)	31 (4–187)	<0.001	-	-	<0.001	<0.001
**Hospitalization days**	4 (0–26)	11 (6–27)	0.001	4 (0–10)	52	18 (0–103)	25 (1–179)	0.001	-	-	<0.001	0.044

Note: *p1* value: The comparison of confirmed H5N1 fatalities and survivors. *p2* value: The comparison of H7N9 fatalities and survivors. *p3* value: The comparison of confirmed H7N9 and H5N1 fatalities. *p4* value: The comparison of confirmed H7N9 and H5N1survivors. We used ANOVA analysis to analyze the average age and median days for a four-group comparison and a T-test to analyze the average age and median days for a two-group comparison. Chi-square (χ^2^) tests were used to compare the distribution of the different variables of qualitative measurements such as gender distribution; a Kruskal-Wallis test was used in the analysis of proportion in the different age groups. The difference is significant between the two groups (*p* < 0.05). CFR = case fatality rate, HPAI = highly pathogenic avian influenza, LPAI = low pathogenicity avian influenza, LBM = live bird markets. “-” = not available.

**Table 2:** Highly pathogenic avian influenza and low pathogenicity avian influenza: An epidemiological and clinical comparison of pediatric fatalities and survivors.

Groups	HPAI (H5N1)			L PAI (H7N9)			*p3*
	Fatalities (n = 97)	Survivors (n = 132)	*p1*	Fatalities (n = 1)	Survivors (n = 42)	*p2*	
**Percentage of total deaths (%)**	33.1 (97/293)	-	-	0.4 (1/265)	-	-	0.030
**CFR (%)**	42.5 (97/228)	-	-	2.4 (1/41)	-	-	<0.001
**Male percentage (%)**	46.4 (45/97)	51.9 (68/131)	0.410	100 (1/1)	45.2 (19/42)	-	0.452
**Median age (Range, (Years))**	6.0 (0.9–15)	4.0 (0.7–15)	<0.001	13	5.0 (0.75–15)	-	0.153
**Median number of days**							
**Days from onset to hospitalization**	6 (2–13)	6 (0–25)	0.963	7	2.0 (0–8)	-	0.008
**Days from onset to confirmation of infection**	10 (3–15)	3 (3–14)	0.034	13	6.5 (1–67)	-	0.025
**Days from onset to antiviral treatment**	7 (0–14)	4 (0–25)	0.044	13	2.5 (0–13)	-	0.045
**Days from onset to outcome**	13 (3–65)	10 (6–20)	0.441	17	10 (5–15)	-	0.905
**Hospitalization days**	7 (1–61)	8 (6–18)	0.596	10	7 (1–14)	-	0.271

Notes: *p1* value: The comparison of confirmed H5NI fatalities and survivors. *p2* value: The comparison of confirmed H7N9 fatalities and survivors. *p3* value: The comparison of confirmed H7N9 and H5N1 survivors. “-”: Not available. For the survivors, the outcome was defined as the day the patient was discharged from hospital. However, for the fatalities, the outcome was defined as the day the patient died from the disease. The age cutoff used for pediatric cases was defined as 0–15 years old.

**Table 3:** Demographic characteristics of clustered H5N1 and H7N9 virus fatalities.

Characteristics	H5N1 Cluster Fatalities			H7N9 Cluster Fatalities		
	Index Cases (n = 43)	Secondary Cases (n = 42)	*p1*	Index Cases (n = 9)	Secondary Cases (n = 6)	*p2*
**Percentage of total fatalities (%)**	14.7% (43/293)	14.3% (42/293)	0.907	3.4% (9/265)	2.3% (6/265)	0.432
**CFR in clustered cases (%)**	100% (43/43)	43.3% (42/97)	<0.001	37.5% (9/24)	20.7% (6/29)	0.176
**Median age (range)**	21 (5–69)	19 (0.75–39)	0.435	56 (37–77)	54 (21–87)	0.872
**Age group (Years)**						
**0–9**	16.3% (7/43)	16.7% (7/42)	0.632	0.0% (0/9)	0.0% (0/6)	0.657
**10–19**	44.2% (19/43)	31.0% (13/42)		0.0% (0/9)	0.0% (0/6)	
**20–29**	14.0% (14/43)	35.7% (15/42)		0.0% (0/9)	16.7% (1/9)	
**30–39**	16.3% (7/43)	16.7%(7/42)		11.1% (1/9)	16.7% (1/9)	
**40–49**	4.7% (2/43)	0.0% (0/42)		11.1% (1/9)	0.0% (0/6)	
**50–59**	2.3% (1/43)	0.0% (0/42)		44.4% (4/9)	33.3% (2/9)	
**Over 60**	2.3% (1/43)	0.0% (0/42)		33.3% (2/9)	33.3% (2/9)	
**Gender**						
**Female**	65.1% (28/43)	52.4% (22/42)	0.233	22.2% (2/9)	33.3% (2/6)	0.634
**Male**	34.9% (15/43)	47.6% (20/42)	0.233	77.8% (7/9)	66.7% (4/6)	0.634
**Comorbidities**	9.3% (4/43)	0.0% (0/42)	0.043	66.7% (6/9)	50% (3/6)	0.622
**Exposure history**						
**Any exposure to poultry**	67.4% (29/43)	45.2% (19/42)	0.039	100% (9/9)	50% (3/6)	0.018
**Common exposure or human case-contact**	0.0% (0/43)	28.6% (12/42)	<0.001	11.1% (1/9)	100% (6/6)	0.001
**Median number of days**						
**Days from onset to hospitalization**	5 (1–8)	5 (2–10)	0.613	5 (2–10)	3 (0–7)	0.305
**Days from onset to confirmation of infection**	11 (7–18)	12 (6–14)	0.089	10 (6–15)	9 (6–13)	0.956
**Days from onset to antiviral treatment**	5 (0–10)	6 (6–12)	0.057	7 (3–12)	7 (3–12)	0.781
**Days from onset to death**	8 (2–22)	9(3–14)	0.450	20 (7–57)	44 (13–85)	0.085
**Hospitalization days**	3.5 (0–16)	4(1–10)	0.406	16 (1–54)	40 (10–83)	0.125

Notes: *p1* value: The comparison of confirmed H5N1 index and secondary fatalities. *p2* value: The comparison of the confirmed H7N9 index fatalities and secondary fatalities. **Any exposure to poultry including:** direct contact; indirect contact; proximity to healthy; sick or dead poultry (including all types of poultry or birds, e.g., chickens, ducks, geese, pet birds, pigeons, etc.); having poultry in the neighborhood; and eating poultry products that have not been properly processed. **Human case contact:** close contact with a confirmed or probable human H5N1/H7N9 case (any time from the day before the onset of illness to when the individual died, or during the period that the individual was hospitalized) in the two weeks before the onset of symptoms. **Common exposure:** including two or more cases of direct or indirect contact with the same poultry or poultry related environments in the two weeks before the onset of symptoms.

**Table 4:** Multivariate logistic regression model assessing odds ratios of risk for death for case-patients infected with highly pathogenic avian influenza (H5N1) and low pathogenicity avian influenza (H7N9) virus.

Variable	H5N1 Cases (N = 390)			H7N9 Cases (N = 323)		
	Value	Odds Ratio (95% CI)	*p* Value	Value	Odds Ratio (95% CI)	*p* Value
**Male sex, no. (%)**	167/390 (42.82)	0.85 (0.06–5.95)	0.465	234/323 (72.4)	0.61 (0.36–1.04)	0.07
**Mean (SD) age, years**	22 (8.5)	2.58 (0.97–3.68)	<0.001	52.08 (20.69)	1.03 (1.02–1.05)	<0.01
**Concurrent health condition, no. (%)**	24/188 (12.77)	4.15 (2.23–12.49)	<0.001	190/323 (58.8)	1.18 (0.71–1.97)	0.52
**Exposure to poultry history, no. (%)**	176/188 (93.62)	1.85 (0.98–7.25)	0.044	189/323 (58.5)	0.73 (0.45–1.18)	0.20
**Median time-to-diagnosis, mo (IQR)**	10 (1–14)	3.55 (2.14–5.78)	0.025	8 (5)	1.01 (0.96–1.06)	0.71
**Median time-to- to antiviral drug treatment , mo (IQR)**	5 (1–14)	1.00 (0.89–1.14)	0.357	6 (4)	1.00 (0.91–1.09)	0.93

Notes: IQR, Interquartile range; CI, Confidence Interval.

## Supplementary Materials

The following are available online at http://www.mdpi.com/1660-4601/14/3/263/s1, Table S1: Summary of the total cases, deaths and case fatality rates (CFR) in highly pathogenic avian influenza viruses H5N1 and H5N6, and low pathogenicity avian influenza viruses H7N9 and H9N2 dates of onset from 1 January 1997 to 30 November, 2016.

## Abbreviations

The following abbreviations are used in this manuscript:

CFR: Case-fatality rate.
ILI: Influenza-like illness.
rRT-PCR: Reverse transcription polymerase chain reaction.
HPAI: Highly pathogenic avian influenza.
LBM: Live bird markets.
LPAI: Low pathogenicity avian influenza virus.
