# Supplementary material for: Comparative Epidemiology of Human Fatal Infections with Novel, High (H5N6 and H5N1) and Low (H7N9 and H9N2) Pathogenicity Avian Influenza A Viruses
Source: Int J Environ Res Public Health. 2017 Mar 4;14(3):263. doi: 10.3390/ijerph14030263 (PMC5369099; doi:10.3390/ijerph14030263)
Supplement: Supplementary file 1 [file ijerph-14-00263-s001.pdf]

International Journal of
Environmental Research and Public Health

MDPI

Supplementary Material: Comparative Epidemiology of Human Fatal Infections with Novel, High (H5N6 and H5N1) and Low (H7N9 and H9N2) Pathogenicity Avian Influenza A Viruses

Zu-Qun Wu, Yi Zhang, Na Zhao, Zhao Yu, Hao Pan, Ta-Chien Chan, Zhi-Ruo Zhang and She-Lan Liu

Table S1. Summary of the total cases, deaths and case fatality rates (CFR) in highly pathogenic avian influenza viruses H5N1 and H5N6, and low pathogenicity avian influenza viruses H7N9 and H9N2 dates of onset from 1 January 1 1997 to 30 November, 2016.

Avian Influenza	Subtype	Countries	Province	No. of cases	No. of deaths	CFR
HPAI	H5N1	Azerbaijan	-	8	5	62.5%
	H5N1	Bangladesh		7	1	14.3%
	H5N1	Cambodia	-	38	29	76.3%
	H5N1	Canada	-	1	1	100.0%
	H5N1	China	-	45	30	66.7%
	H5N1	Djibouti	-	1	0	0.0%
	H5N1	Egypt	-	173	63	36.4%
	H5N1	Indonesia	-	193	161	83.4%
	H5N1	Iraq	-	3	2	66.7%
	H5N1	Laos		2	2	100.0%
	H5N1	Burma	-	1	0	0.0%
	H5N1	Nigeria	-	1	1	100.0%
	H5N1	Pakistan	-	3	1	33.3%
	H5N1	Thailand	-	25	17	68.0%
	H5N1	Turkey	-	12	4	33.3%
	H5N1	Vietnam	-	125	62	49.6%
	**H5N1 Total**		**-**	**638**	**379**	**59.4%**
	H5N6	China	Anhui	1		0.00%
	H5N6	China	Guangdong	6	5	83.33%
	H5N6	China	Hunan	4	2	50.00%
	H5N6	China	Guizhou	1	1	100.00%
	H5N6	China	Hubei	1		0.00%
	H5N6	China	Sichuang	1	1	100.00%
	H5N6	China	Yunnan	2	2	100.00%
	**H5N6 Total**			16	11	68.75%
LPAI	H7N9	China	Zhejiang	197	76	38.6%
	H7N9	China	Guangdong	184	69	37.5%

	H7N9	China	Jiangsu	80	36	45.0%
	H7N9	China	Fujian	63	17	27.0%
	H7N9	China	Shanghai	48	28	58.3%
	H7N9	China	Anhui	32	18	56.3%
	H7N9	China	Hunan	26	11	42.3%
	H7N9	China	Jiangxi	12	1	8.3%
	H7N9	China	Xijinang	10	9	90.0%
	H7N9	China	Shandong	7	3	42.9%
	H7N9	China	Beijing	6	2	33.3%
	H7N9	China	Henan	4	1	25.0%
	H7N9	China	Guangxi	2	1	50.0%
	H7N9	China	Jilin	2	1	50.0%
	H7N9	China	Guizhou	1	1	100.0%
LPAI	H7N9	China	Hebei	1	1	100.0%
	H7N9	China	Hubei	1		0.0%
	**H7N9 Total**			**676**	**275**	**40.7%**
	H9N2	Bangladesh	-	2	0	0.0%
	H9N2	China	-	10	0	0.0%
	H9N2	Egypt	-	3	0	0.0%
	H9N2	Hong Kong SAR	-	6	0	0.0%
	**H9N2 Total**			**21**	**0**	**0.0%**

Notes-: No available.
